# Neuronal mechanism of innate rapid processing of threating animacy cue in primates: insights from the neuronal responses to snake images

**DOI:** 10.3389/fpsyg.2024.1462961

**Published:** 2024-08-29

**Authors:** Tsuyoshi Setogawa, Jumpei Matsumoto, Hisao Nishijo, Hiroshi Nishimaru

**Affiliations:** ^1^System Emotional Science, Faculty of Medicine, University of Toyama, Toyama, Japan; ^2^Research Center for Idling Brain Science, University of Toyama, Toyama, Japan; ^3^Faculty of Human Sciences, University of East Asia, Yamaguchi, Japan

**Keywords:** extrageniculate visual system, single unit activity, monkey, evolution, defense response

## Abstract

To survive in nature, it is crucial for animals to promptly and appropriately respond to visual information, specifically to animacy cues that pose a threat. The subcortical visual pathway is thought to be implicated in the processing of visual information necessary for these responses. In primates, this pathway consists of retina-superior colliculus-pulvinar-amygdala, functioning as a visual pathway that bypasses the geniculo-striate system (retina-lateral geniculate nucleus-primary visual cortex). In this mini review, we summarize recent neurophysiological studies that have revealed neural responses to threatening animacy cues, namely snake images, in different parts of the subcortical visual pathway and closely related brain regions in primates. The results of these studies provide new insights on (1) the role of the subcortical visual pathway in innate cognitive mechanisms for predator recognition that are evolutionarily conserved, and (2) the possible role of the medial prefrontal cortex (mPFC) and anterior cingulate cortex (ACC) in the development of fear conditioning to cues that should be instinctively avoided based on signals from the subcortical visual pathway, as well as their function in excessive aversive responses to animacy cues observed in conditions such as ophidiophobia (snake phobia).

## Introduction

**Figure 1:**
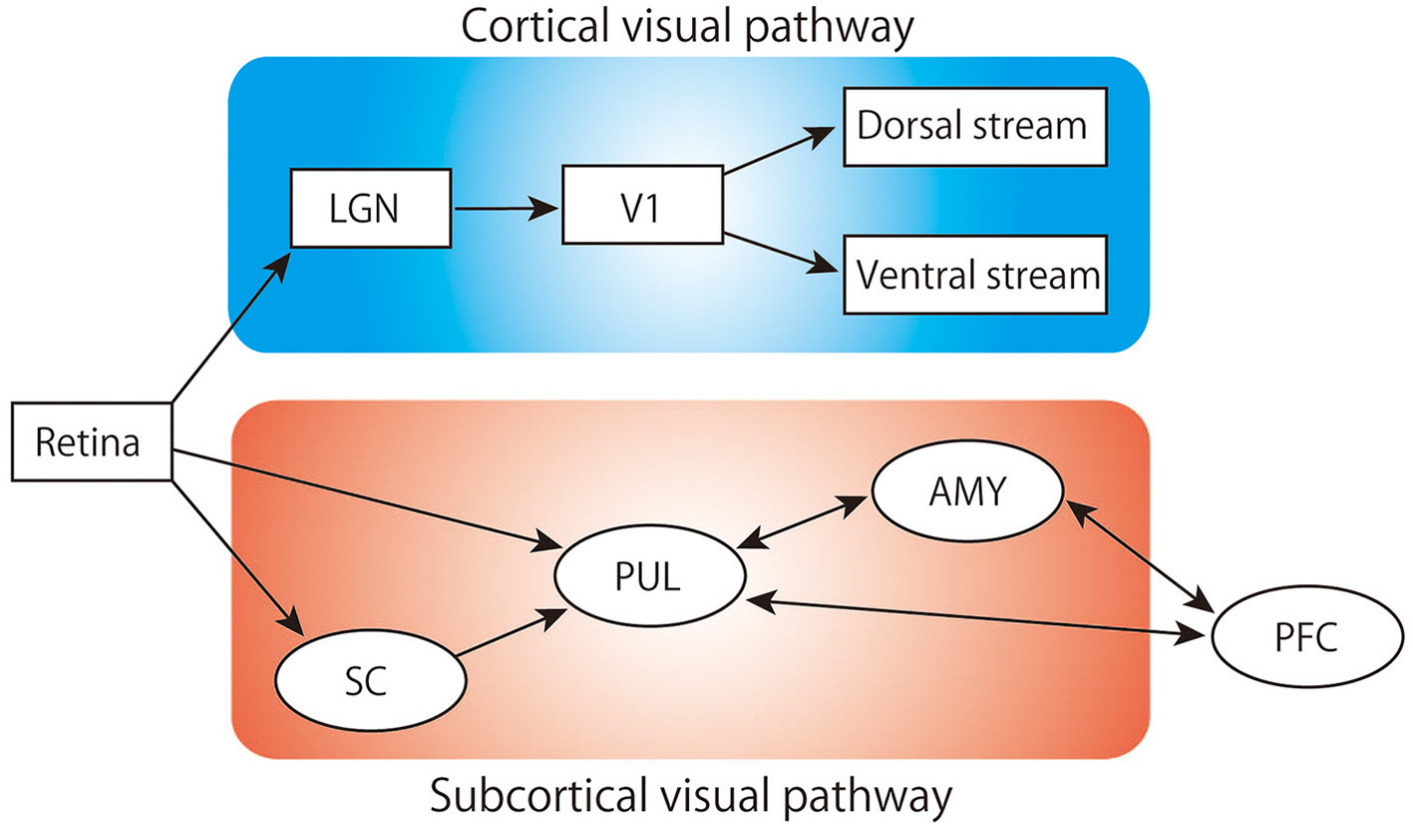
Schematic of the visual pathway. LGN, lateral geniculate nucleus; V1, primary visual cortex; SC, superior colliculus; PUL, pulvinar; AMY, amygdala; PFC, prefrontal cortex.

Rapid defensive responses to animacy cues that indicate a threat (e.g., from predators), such as escape and freezing, are particularly important for avoiding danger and therefore significant for the survival of animals. The neural mechanisms underlying these defensive responses are thought to be primarily innate and shared across various species (LeDoux, 2012). In primates, including humans, information processing through a well-developed visual system is critical for detecting biologically relevant cues. Visual stimuli are conveyed and processed through two major neuronal pathways: the canonical cortical visual pathway and the subcortical visual pathway (Figure 1). The former, also known as the geniculo-striate system, sends retinal information to the visual cortex through the lateral geniculate nucleus (LGN). The information of an object that reached the visual cortex is processed in detail through the temporal cortices for its shape and color, while its spatial location and motion are mainly processed in the posterior parietal region of the cortices (Goodale and Milner, 1992; Kravitz et al., 2013). These pathways play important roles in recognizing, discriminating, categorizing, and processing the movement of visual objects, allowing for the perception and interaction with these objects (Fujita et al., 1992; Tanaka, 1996; Rokszin et al., 2010; Setogawa et al., 2021). The latter pathway, also known as the extrageniculate visual system, consists of the retina-superior colliculus (SC)-pulvinar-amygdala. Similar parallel visual systems can be seen in the avian brain, namely the lemnothalamic (or thalamofugal) and collothalamic (or tectofugal) pathways, which correspond to the mammalian cortical and subcortical pathways, respectively (Clark and Colombo, 2020). In many vertebrates, such as fishes and amphibians, the majority of optic nerve fibers project to the SC (or tectum), and visual circuits involving this pathway are considered to play an important role in innate behaviors (Isa et al., 2021). For example, numerous behavioral studies in frogs have shown that the optic tectum is critical in evoking orienting responses to biologically salient visual stimuli (Ingle, 1973; Masino and Grobstein, 1989). In rodents, about 90% of the retinal ganglion cell axons project to the SC and the circuit including the lateral posterior thalamus (a rodent homolog of the pulvinar) sends animacy cues critical for survival (Carr, 2015; Soares et al., 2017; Isa et al., 2021). In primates, it is estimated that only about 10% of these neurons project to the SC (Perry and Cowey, 1984). Hence, the subcortical visual pathway in primates has long been considered as a vestigial remnant of evolution. However, recent neurophysiological and psychological studies in humans and monkeys have proposed that the subcortical visual pathway is deeply involved in the rapid processing required for the detection of salient visual cues (Soares et al., 2017). The medial part of the frontal cortex, especially the medial prefrontal cortex (mPFC) and the anterior cingulate cortex (ACC), have reciprocal connections with this subcortical visual pathway (Thompson and Neugebauer, 2017; Calderazzo et al., 2021), and this area is involved in the allocation of attention to biologically relevant stimuli (Carretié et al., 2004; Bar et al., 2006). Therefore, it is plausible that the mPFC and ACC receive and integrate swift visual information from the subcortical visual pathway to facilitate or modulate rapid defensive responses. In the first section of this mini review, we summarize the role of the subcortical visual pathway in innate cognitive mechanisms related to threatening animacy cues. In the second section, we discuss the potential role of the mPFC and ACC in fear conditioning to instinctively avoid cues based on signals from the subcortical visual pathway.

### Rapid detection of snakes in the subcortical visual pathway

Snakes, carnivores, and raptors are primary predators of primates during the course of evolution. Among these hunters, sightings or images of snakes evoke significant anxiety and fear in many individuals, suggesting that snakes may have been a particularly salient threat to primate survival in the past (Isbell, 2006). Many behavioral studies have reported that humans and monkeys detect snakes faster than they detect other animals or plants (LoBue and DeLoache, 2008; Masataka et al., 2010; Kawai and Koda, 2016), and that monkeys who have no experience seeing snakes before tend to avoid snake models (Weiss et al., 2015). These findings suggest that primates have evolved to quickly detect and instinctively avoid snakes. To escape from such threats, it is necessary for the animal to promptly react to the relevant visual stimuli even before they reach its consciousness. A likely candidate for orchestrating such rapid response would be the subcortical visual pathway consisting of the SC, pulvinar, and amygdala (Öhman and Mineka, 2001; Johnson, 2005; Isbell, 2009). Recent studies in mice show that this pathway is activated during rapid defensive behaviors such as freezing and escape in response to looming visual stimuli which mimicked a predator, such as birds of prey, approaching from above. Optogenetic activation of SC neurons alone can evoke similar behaviors while inhibition of SC impairs them (Shang et al., 2015, 2018). In monkeys, it has been reported that bilateral lesion of SC impairs the avoidance behavior to snakes (Maior et al., 2011). Furthermore, the population activity of SC neurons in monkeys can discriminate between face-like and non-face-like patterns as early as 50 ms after stimulus onset (Le et al., 2020). This processing seems to occur before the subject recognizes what kind of visual stimuli are presented (Thorpe et al., 1996; Kirchner and Thorpe, 2006). These results suggest that the SC in mammals, including primates, plays a crucial role in rapidly responding to biological salient stimuli that are either socially significant or indicating threats.

The pulvinar is the largest nucleus in the primate thalamus, receiving ascending inputs from the SC and the retina, and projecting to the amygdala and prefrontal regions (Pessoa and Adolphs, 2010; Bridge et al., 2016). One of the established functions of pulvinar is to shift attention to salient visual stimuli while suppressing responses to others, thereby enabling efficient visual information processing (Soares et al., 2017). We previously reported that single unit activity of monkey pulvinar neurons show stronger and faster responses to snake images compared to other images such as faces and hands of monkeys or simple geometric shapes (Van Le et al., 2013; Le et al., 2016). The response latency to snake images in these studies was very short (average latency ≅ 55 ms). Furthermore, presentation of images with only the low spatial frequency components of snake stimuli elicited a similar response as the original images, whereas images with only high spatial frequency components evoked a much reduced response. Interestingly, coarse visual information of faces is also coded at population level as well in pulvinar (Nguyen et al., 2016). These results indicate that the pulvinar is involved in the coarse but rapid processing of snake images transmitted through the subcortical visual pathway.

Recently, we reported that amygdala neurons also exhibit specific responses to snake stimuli (Dinh et al., 2022). In this study, in addition to the four image categories used for the experiment in pulvinar, we included images of non-predators, raptors, and carnivores as well as emotional/neutral human and monkey faces. We not only obtained results similar to those in pulvinar, but amygdala neurons also showed faster and stronger responses to snake images compared to other images with animacy cues. Additionally, the response magnitudes to each stimulus image was positively correlated between the amygdala and pulvinar, which suggests that the amygdala receives information of snake images from pulvinar. Interestingly, when emotional monkey and human faces were presented, stronger and faster responses were observed compared to neutral facial expressions. In human neurophysiological studies, it has also been reported that the subcortical visual pathway responds to low-resolution images of fearful facial expressions (Vuilleumier et al., 2003), and that the amygdala is activated rapidly (40–140 ms) in response to fearful faces (Luo et al., 2010). Additionally, it has been shown that the subcortical visual pathway is activated when fearful facial expressions are presented in the blind field of patients with blindsight due to damage to the primary visual cortex (Morris et al., 2001). These results imply that the subcortical visual pathway processes visual information independently of the cortical visual pathway and contributes to rapid and unconscious processing of emotional visual stimuli in humans as well (Öhman and Mineka, 2001).

### The role of the prefrontal cortex in processing instinctive avoidance cues

It has been proposed that the prefrontal cortex is involved in rapidly responding to aversive visual stimuli and in processing coarse visual images (Kawasaki et al., 2001; Bar, 2003; Kawai and Koda, 2016), with a crucial function in integrating information of sensory input with memory to facilitate recognition. Previous human functional magnetic resonance imaging (fMRI) studies have reported that the mPFC is activated by the presentation of snakes or emotional faces (Nili et al., 2010; Wu et al., 2016), while the ACC is thought to be involved in directing attention to and evaluating salient visual stimuli (Bush et al., 2000). In anatomical studies using monkeys, in the medial part of the frontal cortex, the mPFC including the ACC receive strong projections from the pulvinar and amygdala (Porrino et al., 1981; Romanski et al., 1997). Taken together, these findings indicate that the mPFC and ACC, in cooperation with the subcortical visual pathway, are involved in fast and coarse visual processing to facilitate the detection of evolutionarily conserved predators and emotional faces.

**Figure 2:**
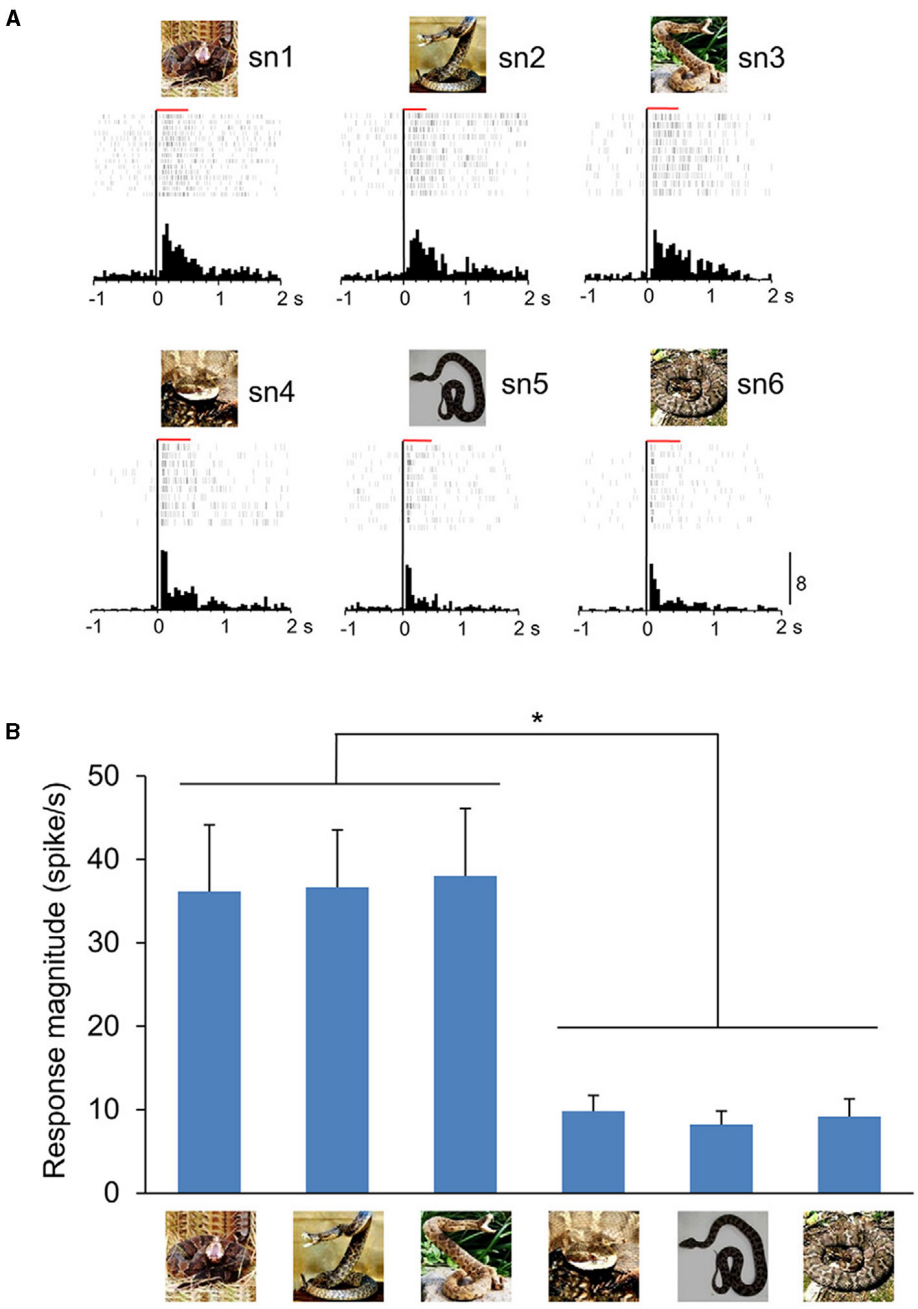
An example of ACC neuron sensitive to snake postures. **(A)** Neuronal responses to each snake image are shown by raster displays and peri-event time histograms. The top three and bottom three graphs indicate the neuronal activity when snakes with striking or non-striking postures were presented, respectively. The red horizontal bars above the raster display indicate the stimulus presentation period (500 ms). Zero on the abscissa indicates the stimulus onset. Calibration at the right bottom of the figure indicates the number of spikes per trial in each bin (Bin width = 50 ms). **(B)** Response magnitudes of this neuron to the six snake images. Histograms indicate mean ± SEM. *p < 0.05. From Dinh et al. (2021).

To test this hypothesis, we recorded from mPFC and ACC in monkeys and presented the same eight categories of images used in the amygdala experiment (Dinh et al., 2018). Remarkably, many neurons in these regions also responded more strongly and quickly to snakes and emotional monkey faces compared to other images. These responses decreased when high-pass filtered visual stimuli were presented but did not decrease with low-pass filtered stimuli (coarse images). Importantly, there was a positive correlation in response magnitude and latency between the pulvinar and both the mPFC and ACC when the same images were presented. The latency of the pulvinar neurons was faster than that of the mPFC and ACC neurons, indicating that the mPFC and ACC receive inputs from the subcortical visual pathway. Furthermore, the rostral part of the ACC showed strong and rapid responses to snakes with striking postures compared to snakes with non-striking postures (Figure 2) (Dinh et al., 2021). Striking postures are generally followed by biting strikes, which makes it crucial to quickly identify a snake's posture to escape attacks from dangerous predators. These results indicate that these cortical regions are likely to be involved in processing threatening animacy cues conveyed through the subcortical pathway. One intriguing hypothesis for their function is that a well-balanced functional interplay of emotion-processing regions such as the amygdala, mPFC, and ACC is important for sufficient fear inhibition and control (Schiller and Delgado, 2010; Sylvester et al., 2012). Disfunction of this interplay may lead to exaggerated anxiety symptoms in response to a specific feared stimulus, often leading to defensive responses that disrupt normal daily activities, as commonly observed in certain phobias. Ophidiophobia (snake phobia), a type of animal-specific phobia, is widely observed worldwide (Fredrikson et al., 1996; Polák et al., 2016). Psychological studies have reported that over 50% of survey participants felt anxiety in response to snakes (Davey, 1994), and 2–3% of participants exhibited reactions similar to ophidiophobia (Polák et al., 2016). A heightened attentional bias toward threat that promotes anxiety has been proposed to be the underlying cause in such phobias (Heeren et al., 2013; LoBue and Rakison, 2013); images of specific animals (such as snakes) are automatically processed in a fear neurocircuitry regardless of attention (Öhman and Soares, 1994), and when the activity for the specific animals exceeds cognitive processing, it captures attention and induces anxiety (i.e., phobia). A human MRI study suggested that abnormalities in amygdala–mPFC connectivity during perception of fearful faces explain phobia severity (Demenescu et al., 2013). Additionally, it has been reported that presenting phobia-related words, e.g., “snake,” to individuals with animal-specific phobias increases activity in the amygdala and ACC compared to healthy controls (Britton et al., 2009). It should be noted, however, that such increase in activity evoked by language-related stimuli is not an innate response and it is unlikely to be caused by inputs from the SC-pulvinar visual pathway described above. According to a functional connectivity analysis based on human fMRI, there was a positive coupling between these regions in phobia groups while negative connectivity was observed in non-phobia groups that might represent fear inhibition in the latter (Stefanescu et al., 2018). These findings suggest that the failure of appropriate control of amygdala activity via the mPFC and ACC when encountering fearful stimuli may lead to exaggerated anxiety symptoms, thereby potentially causing specific phobias.

## Discussion

In this mini review, we summarized the findings indicating functional significance of the evolutionarily conserved subcortical visual pathway for innate mechanisms involved in predator recognition in primates. We also discussed the potential role of the mPFC and ACC, which have reciprocal connections with this pathway, in processing of feared objects as instinctual avoidance signals, as exemplified by ophidiophobia.

The neurocircuitry for the quick detection of threatening animacy cues may be conserved across species. Rodents that have a circuit analogous to the primate subcortical visual pathway, also have regions homologous to the primates' mPFC and ACC (Barthas and Kwan, 2017; van Heukelum et al., 2020). In rodents, in which specific neuronal connections between different brain regions are amply examined by optogenetical approaches, it has been shown that there is a reciprocal connection between the mPFC and amygdala (Huang et al., 2020; Kim et al., 2022), as well as between the ACC and amygdala (Kim et al., 2023). Based on these findings, while neurons in rodents may not elicit a specific response to snakes similar to that in primates, it is conjectured that the subcortical visual pathway-PFC circuit shares a common functional role with the evolutionarily conserved circuits for detecting threats. Therefore, utilizing similar experimental techniques to those employed in rodents may provide further insights into how the subcortical visual pathway, mPFC, and ACC coordinate to elicit rapid defensive responses to innate threats in primates (Raper and Galvan, 2022; Merlin and Vidyasagar, 2023).

During the course of evolution, primates have relied on a well-developed visual system to detect dangerous stimuli. Among many theories regarding the development of the visual system, a particularly interesting one from the point of innate defensive behavior is the “snake detection theory” (Isbell, 2006, 2009). This hypothesis suggests that snakes originated prior to early primates and were their most significant predators, therefore individuals who were adept at visually detecting snakes had a higher chance of survival. Interestingly, studies using electroencephalography on humans have shown that specific neural responses are observed when presenting snake images compared to images of other animals (Van Strien et al., 2014; Bertels et al., 2020). In our previous electrophysiological studies, it was found that neurons in the amygdala, mPFC, and ACC show faster and stronger responses when snake images are presented compared to carnivores and raptors (Dinh et al., 2018, 2022). These results are compatible with the “snake detection theory.” However, it remains unclear which visual aspects of snakes are involved in rapid detection process in the subcortical visual pathway. Some studies have reported that there is a population of pulvinar neurons that are activated by flickering checkerboard patterns (Öhman and Mineka, 2001; Kastner et al., 2004), which closely resemble the scale patterns of snakes. These results suggest that rapid snake detection may also rely on such visual features, but further studies are required to address this point.

The significance of the role of the primate subcortical visual pathway in detection of animacy cues continues to be debated, in spite of the accumulation of neurophysiological evidence in monkeys and humans as described above (Pessoa and Adolphs, 2010; Soares et al., 2017). A recent study in monkeys has reported that the neuronal responses to facial images with short latency in the SC are affected by a pharmacological inhibition of the LGN (Yu et al., 2024), suggesting that inputs from the cortical visual pathway may contribute to such rapid responses in the SC. Since there is no evidence that LGN neurons directly project to the SC in primates, it has been suggested by results obtained from computational modeling that V1 might be the prime candidate to transmit such information to the SC (Yu et al., 2024). It will be of great interest to further examine the contribution of the cortical visual pathway to the rapid detection of animacy cues and its interaction with the subcortical visual pathway in primates. Applying state-of-the-art optogenetic techniques mentioned above might be a powerful experimental tool to address these questions in monkeys (Merlin and Vidyasagar, 2023).

In addition, many of the studies in humans that are referred to in the current review used fMRI, which monitors the blood oxygenation level-dependent (BOLD) signal to visualize the activity of the brain. BOLD signal is known to be an indirect measurement of local neural activity and although it is shown to be related with neuronal firings recorded by electrophysiological methods, it is unlikely to directly reflect their temporal pattern (Logothetis et al., 2001; Drew, 2019). Therefore, the interpretation of human fMRI results and the comparison with single-unit recording in monkeys in particular, requires caution. It has been shown that fMRI measurements in behaving monkeys can be a powerful approach to understand the function of the visual cortex (Tsao et al., 2003; Vanduffel et al., 2014), and considering the difficulty of systematic single-unit recordings in human subjects, further fMRI studies addressing the response to animacy and threating cues in the subcortical visual pathway in monkeys may help considerably to bridge this methodological gap (Passingham, 2009).

At present the pathological mechanisms at circuit level underlying specific phobias in humans including ophidiophobia, remains unclear. It has been proposed that specific phobias may have an evolutionary origin (Mineka and Öhman, 2002; Rakison, 2018). As mentioned above, the subcortical visual pathway is evolutionarily conserved, and considering the specific neuronal responses in this pathway, mPFC, and ACC to snake images, these regions may be involved in the pathogenesis of ophidiophobia. Pharmacological and lesion studies have revealed that the amygdala plays a central role in the acquisition of fear conditioning, formation and storage of fear memories, and their recall (Hitchcock and Davis, 1986; Davis, 1992; Muller et al., 1997). Interestingly, in humans, it is known that fear conditioning is more likely to occur when snake images are used (Öhman and Mineka, 2003). Studies using rodents have reported that neuronal inputs from the ACC are necessary for fear conditioning in the amygdala (Bissière et al., 2008; Jhang et al., 2018). From these findings, it is suggested that fear conditioning to snakes, enhanced via the ACC-amygdala circuit, may lead to exaggerated anxiety contributing to the development of ophidiophobia. Further research in monkeys might provide further insight into how such interaction between cortical and subcortical circuits contributes to the pathogenesis of specific phobias.
